# SEX DIFFERENCES IN FACTORS ASSOCIATED WITH FALLS AMONG COMMUNITY-DWELLING OLDER ADULTS WITH OSTEOARTHRITIS

**DOI:** 10.1093/geroni/igae098.3715

**Published:** 2024-12-31

**Authors:** Aya Yoshikawa, Horng-Shiuann Wu

**Affiliations:** Texas Woman’s University, Dentin, Texas, United States; Michigan State University, East Lansing, Michigan, United States

## Abstract

Osteoarthritis elevates the risk of falling among older adults due to joint pain and stiffness. Research suggests that sleep problems, depression, and fatigue may also relate to falls among those with osteoarthritis, yet sex influences in these associations remain understudied. This study investigated factors associated with falls by sex (male vs. female). A sample of 3,895 community-dwelling older adults with osteoarthritis in the 2016 Health and Retirement Study (2,624 females; 1,271 males) were analyzed using survey-weighted logistic regression. All analyses were controlled for sociodemographic characteristics, including geographic residence, and health-related issues (e.g., balance problems, chronic diseases, and medication use). Most participants were female (64.5%), in their 70s (M = 73.74, SD = 6.4), White (89.0%), had a high school or higher education (89.3%), and non-rural residents (70.9%). For both men and women, balance problems, functional limitations, and cancer increased the risk of falling, while pain, sleep problems, depression, or fatigue were not associated with falls. Among women, being White (OR = 3.87, 95% CI; 1.86, 8.05 compared to Black), rural residence (OR = 1.59, 95% CI: 1.01–2.50 compared to urban residence), and opioid use (OR = 1.60, 95% CI: 1.00, 2.54) increased the risk of falling. Among men, having heart problems (OR = 1.84, 95% CI: 1.24, 2.72) increased the risk of falling. Sex-tailored fall prevention strategies, such as providing rural programs and opioid education for women and increasing awareness and fall education for men with heart problems, are encouraged to support active living among older adults with osteoarthritis.

